# Optimum Installation of Sorptive Building Materials Using Contribution Ratio of Pollution Source for Improvement of Indoor Air Quality

**DOI:** 10.3390/ijerph13040396

**Published:** 2016-04-01

**Authors:** Seonghyun Park, Janghoo Seo

**Affiliations:** 1 The Graduate School of Architecture, Kookmin University, Seoul 02707, Korea; park4mm@nate.com; 2 School of Architecture, Kookmin University, Seoul 02707, Korea

**Keywords:** optimum installation, sorptive building materials, contribution ratio of pollution source, indoor air quality

## Abstract

Reinforcing the insulation and airtightness of buildings and the use of building materials containing new chemical substances have caused indoor air quality problems. Use of sorptive building materials along with removal of pollutants, constant ventilation, bake-out, *etc.* are gaining attention in Korea and Japan as methods for improving such indoor air quality problems. On the other hand, sorptive building materials are considered a passive method of reducing the concentration of pollutants, and their application should be reviewed in the early stages. Thus, in this research, activated carbon was prepared as a sorptive building material. Then, computational fluid dynamics (CFD) was conducted, and a method for optimal installation of sorptive building materials was derived according to the indoor environment using the contribution ratio of pollution source (CRP) index. The results show that a method for optimal installation of sorptive building materials can be derived by predicting the contribution ratio of pollutant sources according to the CRP index.

## 1. Introduction

Reinforcing the insulation and airtightness of buildings and the use of building materials containing new chemical substances has caused indoor air quality problems, such as sick house syndrome, affecting not only the health of residents but also causing economic loss through work efficiency loss. The use of sorptive building materials along with the removal of pollutants, constant ventilation, bake-out, *etc.* are gaining attention in Korea and Japan as methods for improving such indoor air quality problems [1,2,3]. In particular, sorptive building materials have the advantage of reducing the energy load related to the operation of ventilation equipment, as they reduce pollutants in building materials [4].

The development and use of sorptive building materials are increasing, and the test standard widely used in Korea and Japan has been extended to that recommended by the International Organization for Standardization. According to the International Organization for Standardization, the pollutant concentration reducing performance of sorptive building materials is determined according to the mass transfer coefficient of the surface of sorptive building materials, adsorption characteristics (adsorption isotherm) of sorptive building materials, and pollutant spreading resistance (molecular) diffusion of sorptive building materials. In particular, to conduct an accurate performance evaluation of sorptive building materials according to the chamber method, it is important to recreate the conditions of the actual indoor environment by maintaining the air current of the surface of the test sample. In addition, the International Organization for Standardization states that maintaining environmental variables, including temperature, relative humidity, amount of ventilation, loading factor, and mass transfer coefficient, is essential for comparing sorptive building materials [5].

On the other hand, reviewing and validating previous studies on the performance of sorptive building materials to reduce pollutant concentration and the human body’s ability to reduce pollutant inhalation volume in a real-scale space are necessary. Experiments on the human body regarding the pollutant inhalation volume entail several risks. In addition, once sorptive building materials are installed, it is difficult to move them, which means that the optimal installation plan should be reviewed in advance so that the pollutant concentration reducing performance of the sorptive building materials can be maximized. Computational fluid dynamics (CFD) analysis does not cause any risks resulting from the experiment and has the advantage of deriving an optimal installation plan for sorptive building materials by reviewing the influence of pollutants and indoor environmental factors at the design stage.

In this study, an experiment on the pollutant reducing performance of activated carbon, which has been proven to have a significant effect on reducing indoor pollutant concentration, was conducted and the sustainability of the performance was analyzed. In addition, based on these results, CFD analysis was conducted to evaluate the concentration of pollutants around an occupant’s breathing area according to the air diffuser method inside the space resulting from the installation of sorptive building materials and the posture of the occupant, and methods for optimal installation of sorptive building materials for reducing pollutant inhalation volume of an occupant were reviewed using the CRP 2 * index.

## 2. Performance of Sorptive Building Materials

**Figure 1:**
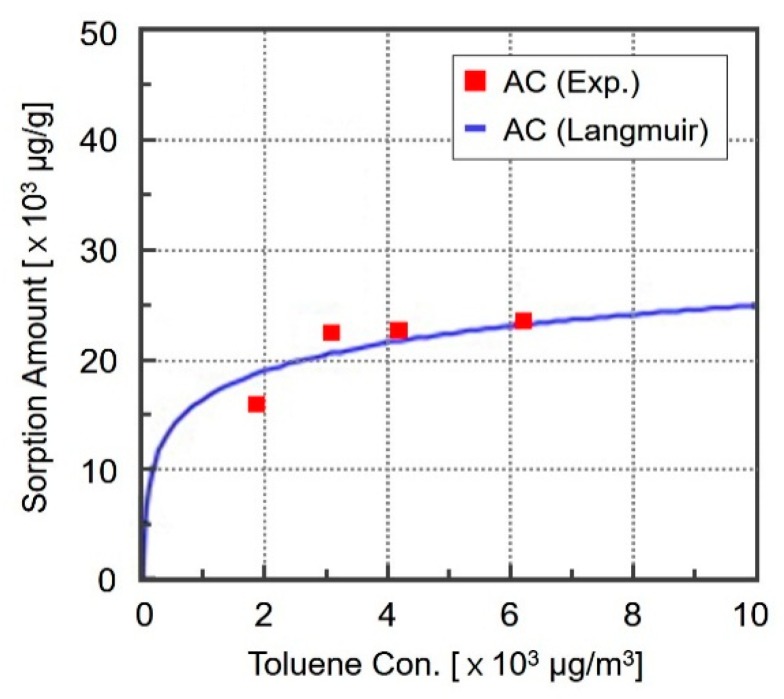
Adsorption isotherm of activated carbon with respect to toluene.

The pollutant reducing performance of activated carbon, selected as the sorptive building material in this study, was evaluated according to the test method suggested in ISO 16000-23, 24, and the results are shown in Table 1 and Figure 1.

A previous study on the pollutant adsorption performance of activated carbon (AC) using a boundary layer-type small test chamber (BLTSTC) showed that when toluene was injected at a concentration of 280 μg/m^3^, the total pollutant inhalation volume detected after 7 days was 9.6 μg/g, which indicates that the adsorption volume was not that significant [4,6]. However, the adsorption isotherm of Figure 1 shows that the adsorption volume of toluene by the AC increased as the concentration of toluene increased. This means that when the concentration of indoor toluene is high, a corresponding improvement in toluene adsorption performance can be expected. Accordingly, in this research, activated carbon was used as the sorptive building material and toluene was set as the pollutant to be adsorbed.

## 3. Outline of CFD Analysis

### 3.1. Geometry and Conditions of Case

**Figure 2:**
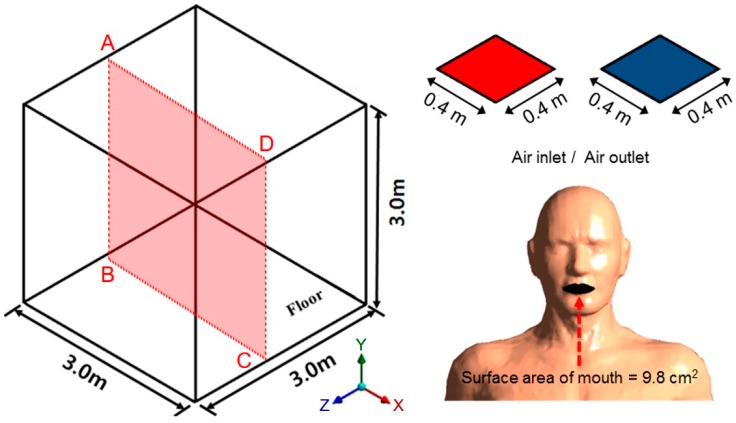
Model used for CFD analysis.

The CFD analysis model for reviewing the diffusion and adsorption of pollutants in the indoor space is shown in Figure 2. Mechanical ventilation was operated in a space measuring 3 m in length, width, and height, and the area of the inlet and outlet was identically set to 0.16 m^2^. To review the thermal diffusion and air distribution near the surface according to the heat of an occupant, a human body structure similar to a real body was used. The size of the inlet of the applied human body model was about 9.8 cm^2^, based on data from previous studies [7,8].

**Figure 3:**
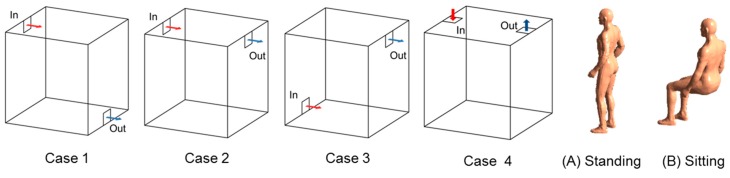
Air diffuser types and types of occupant posture in the case study.

In addition, a case study was conducted to determine the pollutant concentration reducing effect of sorptive building materials and an optimal installation plan for sorptive building materials considering four types of air diffuser methods and two types of occupant postures, as shown in Figure 3; the corresponding case conditions are shown in Table 2.

### 3.2. Numerical Model

This study used the species transport model for CRP calculation. The gas inside the space consisted of three types, all of which included air gas (representing fresh air) and the pollutant toluene. Accordingly, each gas was assumed to be an incompressible ideal gas with temperature dependence. For the turbulence model, the re-normalization group (RNG) k-epsilon turbulence model was used, considering natural convection by buoyancy. Equations (1)–(3) show the equations of continuity, motion, and energy, respectively:

(1) $$\frac{\partial \varrho }{\partial t}+\nabla \cdot \left(\varrho u\right)=0$$

(2) $$\frac{\partial \left(\varrho u\right)}{\partial t}+\nabla \cdot \left(\varrho uu\right)=-\nabla p+\varrho g+\nabla \cdot \left(\mu \nabla u\right)-\nabla \cdot \tau_{t}$$

(3) $$\frac{\partial \left(\varrho e\right)}{\partial t}+\nabla \cdot \left(\varrho eu\right)=\nabla \cdot \left(k_{eff}\nabla T\right)-\nabla \cdot \left(\underset{i}{\sum }h_{i}j_{i}\right)$$

where 𝜚 is density of fluid, *t* is time, *u* refers to fluid velocity vector, *p* is pressure, g is vector of gravitational acceleration, μ is molecular dynamic viscosity, *e* is specific internal energy, *k_eff_* is effective heat conductivity, *T* stands for fluid temperature, *h_i_* refers to specific enthalpy of fluid, and *j_i_* is mass flux of the *i*-th constituent. The last term on the right hand side of Equation (2) is the divergence of the turbulence stresses (Reynolds stresses), *𝜏_t_* accounts for auxiliary stresses due to velocity fluctuations.

For the CRP calculation, it is necessary to calculate the quantitative amount of pollutants from each pollution source. Therefore, an ID was given to each component gas, and the partial differential transport equations of each gas that passed through the control volume (CV) of the 3D space are given by Equation (4) [9]:

(4) $$\frac{\partial Y_{i}}{\partial t}+\nabla \cdot \left(\varrho Y_{i}u\right)=-\nabla \cdot j_{i}$$

where *Y_i_* is mass fraction of the *i*-th air constituent. Due to the reasonably low thermal parameters (pressure and temperature) of air, it can be treated as a rarified mixture.

The initial toluene concentration, before the introduction of pollutants to the target space, was assumed to be C_a_ = 0. This was set to rule out changes in the sorptive performance of the sorptive building materials caused by any variable other than the pollutants.

This study evaluated the improvement in pollutant inhalation rate by occupants after the application of sorptive building materials using the CRP index proposed by Hayashi *et al.* [10]. The CRP is classified into CRP 1 and CRP 2. Among them, CRP 2 is an index indicating how much the pollutant generated from each pollution source contributes to the total pollutant inhalation volume of the occupant. Thus, the sum of the contribution ratio of each pollution source affecting the indoor occupant is always 100%, and is calculated by the following Equation (5):

(5) $$CRP\;2_{i}=\;\frac{q_{i}}{q_{total}}\times 100\%,\;q_{total}=\underset{i}{\sum }q_{i}$$

where *q_i_* is the pollutant inhalation volume (g/h) of the occupant from source *i* and $\sum_{i}q_{i}$ is the total pollutant inhalation volume (g/h) of the occupant according to each source. CRP 2 is an index indicating the contribution ratio of each pollution source with respect to the inhalation volume of the generated pollutant by a human body and has a relative value. Thus, under the assumption that fresh outdoor air is supplied into the indoor space through the inlet at a constant air volume, it becomes possible to evaluate the concentration reducing effect of sorptive building materials based on the ratio of fresh outdoor air to the total human body inhalation volume by calculating CRP 2 including the influence of inflow air. Thus, the contribution ratio including fresh air was defined as CRP 2 * in this research, and the optimal installation plan for sorptive building materials was derived using this rate.

### 3.3. Conditions of CFD Analysis

The CFD analysis conditions used for calculating CRP 2 * are shown in Table 3. The velocity of air flowing through the inlet was 1.0 m/s. For the mesh of the object in 3D space, a triangular mesh was created around the human body model and hexa mesh was created for other spaces in consideration of symmetry. In order to increase the convergence of natural convection according to buoyancy, PRESTO was used as a solution for the continuity equation, and the heating volume of the human body model and the respiration volume at the normal state were applied based on the existing literature [7]. Additionally, this research does not account for the impact of humidity.

## 4. Results of CFD Analysis

### 4.1. Air Distribution of Object Space

**Figure 4:**
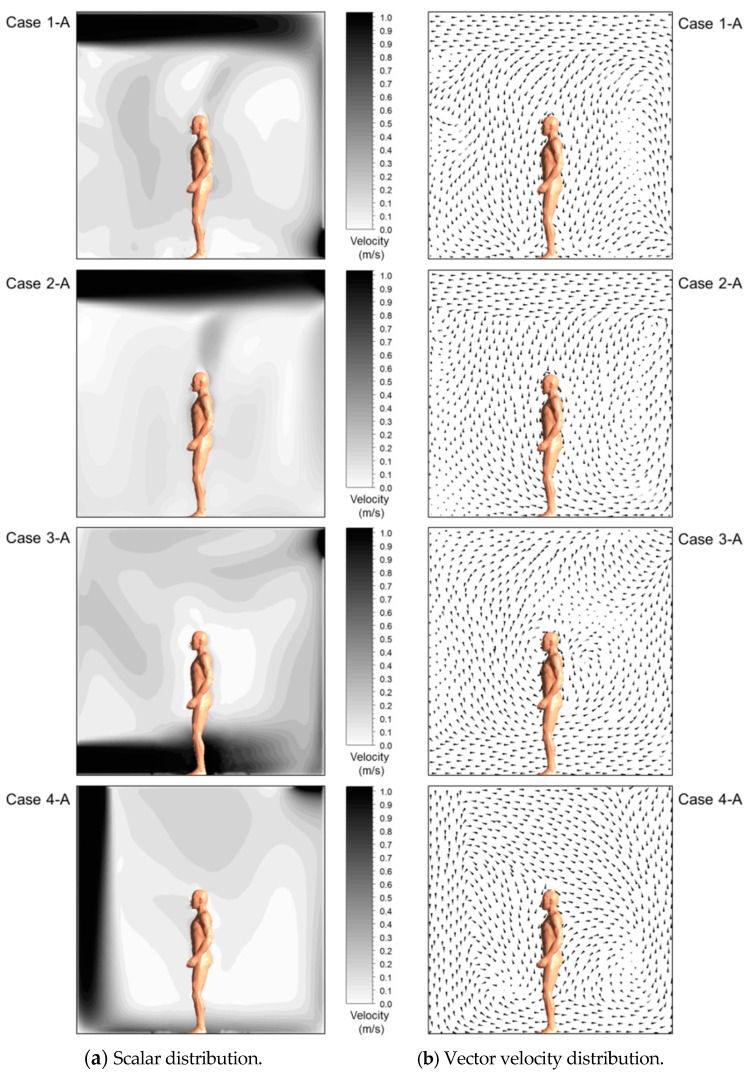
Air flow (Plan ABCD in Figure 2). (**a**) Scalar distribution; (**b**) Vector velocity distribution.

**Figure 5:**
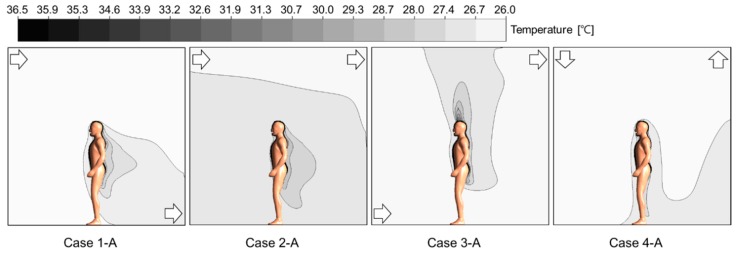
Temperature distribution around the human model (Plan ABCD in Figure 2).

Figure 4 shows the air distribution maps for each air diffuser condition when the occupant was standing. Regardless of the case, the velocity of the air current around the inlet was observed to be the highest. In Case 2, where the inlet and outlet were placed on the upper side, the velocity of airflow was somewhat low because the introduced air current escaped through the outlet without being distributed in the air flow. In addition, it was revealed that temperature changed around the occupant’s body surface due to the heat generated by the occupant, as illustrated in Figure 5.

### 4.2. Contribution Ratio of Each Pollution Source on Indoor Occupants

**Figure 6:**
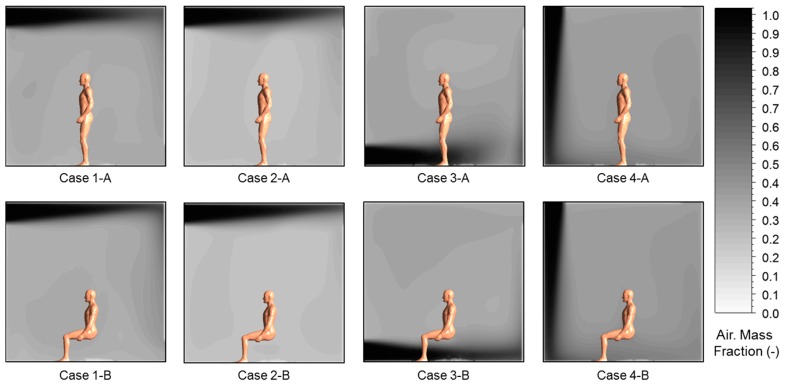
Air Mass Fraction distribution according to fresh outdoor air for each case (Plan ABCD in Figure 2).

**Figure 7:**
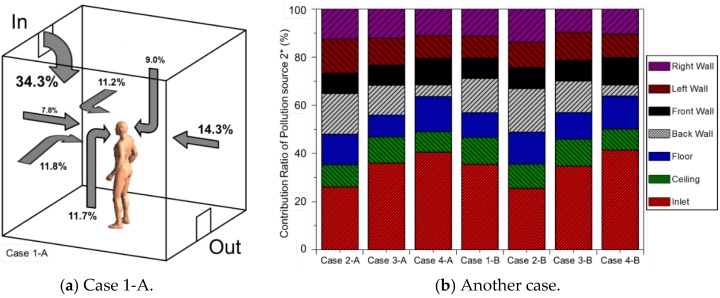
CRP 2 * calculation results for each case. (**a**) Case 1-A; (**b**) Another case.

In this research, toluene was used as the pollution source and CRP 2 * was calculated by assigning a pollutant concentration condition (mass fraction = 1.0) to each wall’s surface. Figure 6 shows the mass fraction distribution of fresh outdoor air introduced to the indoor space through the inlet. The indoor concentration diffusion of the outdoor air was highly dependent on the velocity of the air current and thus showed a tendency similar to the air current distribution. The contribution ratio, calculated in order to consider the optimal installation plan for sorptive building materials around the occupant, is shown in Figure 7. The sum of the percentage of CRP 2 * in each case was set to 100%, and the change in the contribution ratio of each pollution source was observed to be larger according to the air diffusion method rather than the posture of the occupant. This is deemed to be because the breathing areas formed by the occupant while standing or sitting do not have a large effect. However, in Case 4, where the air supply and exhaust were at the ceiling, the ratio of fresh outdoor air to the respiration volume of the occupant was the highest. This means that in subject spaces where the length, height, and width are identical to each other and a constant outdoor air volume is supplied indoors, ceiling air supply systems cause the highest amount of fresh outdoor air to be present in the breathing area of the occupant. In Case 2, the age of the air was lowest as the fresh outdoor air was discharged through the outlet without being disturbed by the flow of air current, and therefore the contribution ratio of the fresh outdoor air to the respiration volume of the indoor occupant was about 26.3%.

### 4.3. Change in Contribution Ratio per Indoor Pollution Source according to the Installation of Sorptive Building Materials

**Figure 8:**
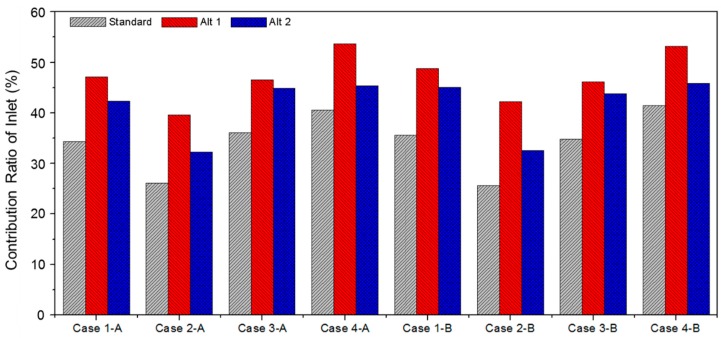
CRP 2 * for each case. Standard: Before the installation of sorptive building materials. Alt 1: Sorptive building materials were installed at the pollution source with the highest contribution ratio. Alt 2: Sorptive building materials were installed at the pollution source with the lowest contribution ratio.

Calculating the contribution ratio (CRP 2 *) of each pollution source before installing sorptive building materials showed that the contribution ratio of the pollutant generated from the side vertically closest to the inlet turned out to be the highest in every case. Accordingly, it was confirmed that the floor in Case 4 and the back wall in the remaining cases had the highest contribution rate; the pollution source with the highest contribution ratio was set as the object while reviewing the change in the contribution rate of the fresh outdoor air according to the sorptive building materials for determining the optimal installation plan for sorptive building materials. In addition, as a control group, sorptive building materials were installed at the pollution source with the lowest contribution ratio (Alt 2). The results from both cases were compared to each other. Figure 8 shows the change in the contribution ratio of the fresh outdoor air according to the installation of sorptive building materials on the applied sides. In the case where sorptive building materials were installed at the pollution source with the highest contribution ratio (Alt 1) before the installation of sorptive building materials (Standard), the ratio of the fresh outdoor air to the respiration volume of the occupant increased from 25.6% to a maximum of 42.3%. When compared to the control group, the contribution ratio of the fresh outdoor air increased when compared to the ratio before the installation of the sorptive building materials, and the case where sorptive building materials were installed in the place with the highest pollution source contribution ratio showed the best pollutant concentration reducing effect around the occupant.

## 5. Conclusions

In this study, optimal installation plans for sorptive building materials were reviewed to reduce pollutant inhalation volumes of indoor occupants using the corrected CRP 2 * index among other CRP indexes. Reviewing the contribution ratio of each pollution source according to the air diffuser method and the posture of the occupant showed that it is possible to select an air diffuser method to reduce the respiration volume of pollutants by occupants to a minimum, regardless of the installation of sorptive building materials, by identifying the age of the fresh outdoor air. In addition, CRP 2 *, which is the contribution ratio of a pollution source, was calculated and a method for optimal application of sorptive building materials, a passive method for reducing pollutant concentration, was suggested. Activated carbon, which adsorbs toluene excellently, was chosen as the sorptive building material to validate its effectiveness; for this, a control group was prepared and analyzed, and the results were compared. The comparison showed that the installation of sorptive building materials based on CRP 2 * had the best pollutant concentration reducing effect. Thus, an optimal installation plan for sorptive building materials targeting occupants or a certain space can be derived by calculating CRP 2 *.

For this research, it was assumed that the performance of the sorptive building materials is high, regardless of the indoor environment. For future research, however, the change in performance of sorptive building materials will be reviewed while accounting for the humidity and moisture.

## Figures and Tables

**Table 1:** Results of pollutant concentration reducing performance of activated carbon.

Supply Gases	Supply Concentration (μg/m^3^)	Exhaust Concentration (μg/m^3^)	Sorption Flux (μg/m^2^·h)	Equivalent Ventilation Rate per Unit Area (m^3^/h·m^2^)	Total Sorption Value for Toluene after 7 Days (μg/g)
Toluene	280	48	52	1.1	9.6
Ethyl benzene	305	55	56	1.0	-
P-xylene	104	18	19	1.1	-
Styrene	300	50	55	1.1	-

**Table 2:** Case for CFD analysis.

Posture	Air Diffuser			
	Case 1	Case 2	Case 3	Case 4
(A) Standing	Case 1-A	Case 2-A	Case 3-A	Case 4-A
(B) Sitting	Case 1-B	Case 2-B	Case 3-B	Case 4-B

**Table 3:** Conditions for CFD analysis.

Turbulent Flow Model	RNG *k-ε* Model
Number of Meshes	Around 3,000,000
Inflow Boundary	$U_{\;in}=\frac{1m}{s},\;k_{in}=\frac{3}{2}{\left(U_{y,\;in}\times 0.05\right)}^{2},\;Temperature=26$ °C $\varepsilon_{in}=C_{u}\times \frac{k_{in}{}^{\frac{3}{2}}}{L_{in}},\;L_{in}=\frac{1}{7L_{o}},\;L_{o}=0.4\;m$
Outflow Boundary	$U_{out,mouth}=outflow\;$ (Mass flow conservation) Flow rate weighting, outlet = 0.99, mouth = 0.01
Wall Boundary	No-slip
Breath Boundary	14.4 L/min (Human standard)
Flux Boundary	$H_{human}=22.8\;W/m^{2},$ (Heat transfer rate of person = 33.8 W)

## Abbreviations

The following abbreviations are used in this manuscript:

CFD: computational fluid dynamics
CRP: contribution ratio of pollution source
AC: activated carbon
BLTSTC: boundary layer-type small test chamber
